# Study of the Imbibition Phenomenon in Porous Media by the Smoothed Particle Hydrodynamic (SPH) Method

**DOI:** 10.3390/e24091212

**Published:** 2022-08-29

**Authors:** Jie Liu, Tao Zhang, Shuyu Sun

**Affiliations:** Computational Transport Phenomena Laboratory, Physical Science and Engineering Division (PSE), King Abdullah University of Science and Technology, Thuwal 23955-6900, Saudi Arabia

**Keywords:** SPH method, two-phase, porous media

## Abstract

Over recent decades, studies in porous media have focused on many fields, typically in the development of oil and gas reservoirs. The imbibition phenomenon, a common mechanism affecting multi-phase flows in porous media, has shown more significant impacts on unconventional reservoir development, where the effect of the pore space increases with decreased pore sizes. In this paper, a comprehensive SPH method is applied, considering the binary interactions among the particles to study the imbibition phenomenon in porous media. The model is validated with physically meaningful results showing the effects of surface tension, contact angle, and pore structures. A heterogeneous porous medium is also constructed to study the effect of heterogeneity on the imbibition phenomenon; it can be referred from the results that the smaller pore throats and wetting surfaces are more preferred for the imbibition. The results show that the SPH method can be applied to solve the imbibition problems, but the unstable problem is still a sore point for the SPH method.

## 1. Introduction

The two-phase problem is common in the academic and engineering fields [1,2]. For example, the flooding processes in the development of petroleum, which include liquid flooding and gas flooding [3,4], are usually accompanied by multi-phase problems. In the oil and gas reservoir, porous media are occupied by the liquid and gas phases with the states of liquid bridges and clusters, and the pore size ranges from nanometers to micrometers [5]; accordingly, the two-phase problem is the key point in the development of reservoirs. Furthermore, the cohesion and the contact angle are always the main research points academically [6].

A number of methods have been used to handle the two-phase problem due to their applicability [7,8,9,10,11]. The non-linear partial differential equation of the two-phase and incompressible fluid was proposed and applied in a porous media [12]. The finite volume method was applied to the two-phase flow in a fractured porous media with fully implicit discretization [13]. The finite element method was also developed for the two-phase immiscible flow problems [14,15].

Apart from the mesh method, the particle method, such as the molecular dynamics, was also applied to the multi-phase problems, revealing the mechanism of phase behaviors at the atomic scale [16,17,18,19]. The smoothed particle hydrodynamics (SPHs) method, a mesh-free method, is fully particle discretized [20], which is good at dealing with the free surface and large deformation problems [6,21]. If the gas phase is taken into consideration based on the free surface problem, it turns into a two-phase problem [22,23].

The SPH method is applied for multi-phase problems using several computational fluid dynamics techniques [10,24,25,26]. For example, the technique of interface tracking between different fluid phases is usually carried out by the color function, and the relation between the surface tension and curvature is controlled by the Young–Laplace equation [27,28]. In the unconventional reservoir, such as the shale reservoir, the pore size is extremely small; as a result, the effects of the micro-confined space cannot be ignored [29,30,31]. Abdolahzadeh et al. [32] studied the mixing processes for the two-phase flow in a single channel with various structures by the meshless SPH method. Tartakovsky et al. [33] studied the mineral precipitation and reaction flow in porous media using the SPH method. They found that the SPH method was good at studying the flow and transport behaviors in pore-scale space. Bui et al. [34] developed the SPH method by coupling the behaviors of the fluid and solid phases in porous media, and the results show that the two-phase SPH method is promising for coupled problems. Kazemi et al. [35] used the spatial averaging method to obtain the mass and momentum conservation equations for comparative research of previous studies. In this case, the pairwise force SPH method has been proposed [36,37], but further studies of its application and validation are still needed.

In this work, the SPH method, which considers the effect of the interaction force between particles, is applied to imbibition problems of the gas and liquid phases innovatively. The homogeneous and inhomogeneous porous media are built, where the particles of the gas and liquid phases are filled as the shapes of bridges and clusters. The sensitivity of the porous media’s structure, the pore size, and the contact angle are also examined.

## 2. Methodology

### 2.1. The Governing Equations

In this work, the weakly compressible fluid is adopted, and the non-linear term in the momentum equation is not taken into consideration [38,39]. The equation of state is needed to calculate the pressures as follows [40]:

(1) $$\frac{d\rho }{dt}=-\rho \nabla \cdot u$$

(2) $$\rho \frac{du}{dt}=-\nabla p+\nabla \cdot \left(\mu \left(\nabla u+\nabla u^{T}\right)\right)+g+F^{S}$$

(3) $$p=p_{eq}\frac{n}{n_{eq}},$$

where the ρ denotes the density of the fluid, the u denotes the fluid velocity, the **g** denotes the gravity acceleration, the **F***^S^* denotes the surface tension term, the *p_eq_* denotes the pressure in the equilibrium state, the *n_eq_* denotes the number density in the equilibrium state, the *p* denotes the pressure of the fluid, and the *n* denotes the number density of the fluid. The Young–Laplace equation is adopted to build the sharp interface model as follows [27,40,41]:

(4) $$\left(p_{l}-p_{g}\right)n=\left(\tau_{l}-\tau_{g}\right)\cdot n+k\sigma n$$

(5) $$\sigma_{lg}\cos\theta_{e}+\sigma_{sl}=\sigma_{sg},$$

where the $p_{l}$ and $p_{g}$ denote the pressures of the liquid and gas phases, respectively; the $\tau_{l}$ and the $\tau_{g}$ denote the viscous stress tensors of the liquid and gas phases, respectively; the n denotes the normal unit vector perpendicular to the interface; the σ denotes the surface tension coefficient; and the $\theta_{e}$ denotes the equilibrium contact angle.

### 2.2. The SPH Model

The SPH method, which is meshless, is carried out by the kernel function approximation and particle approximation as follows:

(6) $$A\left(r\right)\approx \int A\left(r^{\prime }\right)W\left(r-r^{\prime },h\right)dr^{\prime }$$

(7) $$A\left(r\right)\approx \sum_{b}m_{b}\frac{A_{b}}{\rho_{b}}A\left(r^{\prime }\right)W\left(r-r_{b},h\right),$$

where the A(r) denotes the field function; the *W* denotes the kernel function; the r denotes the distance between particles; the *h* denotes the smooth length; the $m_{b}$, $\rho_{b}$, and $A_{b}$ denote the mass, density, and field function of particle *b,* respectively. According to Equations (6) and (7), the differential operators can be discretized in the SPH forms as follows:

(8) $$\nabla A_{a}\approx \underset{b}{\sum }m_{b}\frac{A_{b}}{\rho_{b}}A_{a}\nabla W_{ab}$$

(9) $$\nabla \cdot A_{a}\approx \underset{b}{\sum }m_{b}\frac{A_{b}}{\rho_{b}}A_{a}\cdot \nabla W_{ab}$$

(10) $$\nabla \times A_{a}\approx \underset{b}{\sum }m_{b}\frac{A_{b}}{\rho_{b}}A_{a}\times \nabla W_{ab},$$

where $W_{ab}=W_{a}-W_{b}$. By balancing the coding complexity and computational efficiency, the cubic spline kernel function is adopted as follows [20]:

(11) $$W\left(r,h\right)=\sigma_{d}\{\begin{array}{c} 6\left(q^{3}-q^{2}\right)+1,\;\;\;\;0\leq q\leq 0.5\;\;\\ \;\;\;2{\left(1-q\right)}^{3},\;\;\;\;\;\;\;0.5<q\leq 1\;\;,\;\\ 0,\;\;\;\;\;\;\;\;\;\;\;\;q>1\;\; \end{array}$$

where q=‖r‖/h, the $\sigma_{d}$ denotes the normalization factor of the kernel function, $\sigma_{1D}=4/\left(3h\right)$, $\sigma_{2D}=40/\left(7\pi h^{2}\right)$, and $\sigma_{3D}=8/\left(\pi h^{3}\right)$. Therefore, the continuity equation of weakly compressible fluid can be written in the form of SPH discretization as follows [41]:

(12) $$\frac{d\rho_{a}}{dt}=\underset{b}{\sum }m_{b}u_{ab}\cdot \nabla_{a}W_{ab},$$

where $u_{ab}=u_{a}-u_{b}$ and $\nabla_{a}W_{ab}=-\nabla_{b}W_{ab}$. However, in the momentum equation, it is not a good choice to use the direct discretization form of the pressure gradient since the symmetric form is more stable for the multi-phase problem [6,42], as written in Equation (13).

(13) $${\left(\frac{1}{\rho }\nabla p\right)}_{a}=\sum_{b}m_{b}\left(\frac{p_{a}+p_{b}}{\rho_{a}\rho_{b}}\right)\nabla_{a}W_{ab}.$$

By using the divergence operator and the discretization of the SPH method, the viscosity term can be written as follows [43,44]:

(14) $${\left(\frac{\mu }{\rho }\nabla^{2}u\right)}_{a}=\underset{b}{\sum }m_{b}\frac{\left(\mu_{a}+\mu_{b}\right)r_{ab}\cdot \nabla_{a}W_{ab}}{\rho_{a}\rho_{b}\left(r_{ab}^{2}+0.01h^{2}\right)}u_{ab},$$

where the term $0.01h^{2}$ is used to avoid the singularities [45]. To handle the problem of the gas and liquid phases, the pairwise force is calculated in the surface tension term, where the attractive and repulsive forces can be addressed as follows [46]:

(15) $$F_{ab}^{s}=\{\begin{array}{c} -s_{\alpha \beta }r_{ab}\left[A\Psi_{\varepsilon_{0}}\left(r_{ab}\right)+\Psi_{\varepsilon }\left(r_{ab}\right)\right]\\ 0 \end{array}\;\;\;\;\begin{array}{c} r_{ab}\leq h\\ r_{ab}>h \end{array}\;,\;$$

where the $F_{ab}^{s}$ denotes the interfacial tension force between particles *a* and *b* and the $s_{\alpha \beta }$ denotes the strength coefficient of the interaction. For the two-dimensional cases, $\varepsilon =\frac{h}{3.5}$, $\varepsilon_{0}=\frac{\varepsilon }{2}$, $\Psi_{\varepsilon }\left(r_{ab}\right)=e^{\frac{r_{ab}^{2}}{2\varepsilon^{2}}}$, and $A={\left(\frac{\varepsilon }{\varepsilon_{0}}\right)}^{3}$. The two-phase problem in this study is immiscible; thus, the particles in the same phase need a larger interaction force, and the strength coefficients can be calculated as follows [46,47]:

(16) $$\{\begin{array}{c} s_{\alpha \alpha }=s_{\beta \beta }=0.5n^{-2}{\left(\frac{h}{3}\right)}^{-5}\frac{\sigma }{\lambda }\;\\ s_{s\alpha }=0.5n^{-2}{\left(\frac{h}{3}\right)}^{-5}\frac{\sigma }{\lambda }\left(1+0.5\cos\theta \right)\\ s_{s\beta }=0.5n^{-2}{\left(\frac{h}{3}\right)}^{-5}\frac{\sigma }{\lambda }\left(1-0.5\cos\theta \right) \end{array}\;\;$$

where the n denotes the average number density of the fluid, the σ denotes the surface tension coefficient, and $\lambda =\frac{3}{4\pi^{2}}\left(2^{7}-3^{2}\times 2^{4}\pi^{2}+3^{3}\pi^{4}\right)$. Therefore, $s_{\alpha \alpha }=s_{\beta \beta }=s_{s\alpha }=s_{s\beta }$ if the contact angle is 90º, which suggests that the neutral wetting condition can be obtained. The boundary conditions are as follows [21]:

(17) $$F_{i}^{bound}=\overset{N_{bound}}{\underset{j=1}{\sum }}f_{ij}^{bound}$$

(18) $$f_{ij}^{bound}=\{\begin{array}{cc} -\left[U_{max}^{2}\frac{\min\left(\left(u_{i}-u_{j}\right)\cdot {\hat{n}}_{j},-1\right)W_{ij}H_{ij}{\hat{n}}_{j}}{\left|r_{ij}\cdot n_{j}\right|}\right],\;\; & \left(u_{i}-u_{j}\right)\cdot {\hat{n}}_{j}<0\\ 0,\;\; & \left(u_{i}-u_{j}\right)\cdot {\hat{n}}_{j}>0 \end{array}$$

where the *i* denotes the index of the fluid particle, the *j* denotes the index of the solid particle, the $u_{i}$ denotes the fluid velocity, the $u_{j}$ denotes the solid velocity, and the ${\hat{n}}_{j}$ denotes the normal vector for the solid particle *j*. The solid particles’ velocity and pressure are obtained as follows:

(19) $$u_{j}=-\frac{\sum_{i}^{N_{f}}u_{i}W_{ij}}{\sum_{i}^{N_{f}}W_{ij}}$$

(20) $$p_{j}=\frac{\sum_{i}^{N_{f}}p_{i}W_{ij}+\left(g-b_{j}\right)\cdot \sum_{i}^{N_{f}}\rho_{i}r_{ij}W_{ij}}{\sum_{i}^{N_{f}}W_{ij}}$$

where the $N_{f}$ denotes the number of fluid particles, the $N_{bound}$ denotes the number of solid particles, and the $b_{j}$ denotes the prescribed acceleration for solid particles.

### 2.3. The Relaxation of the Solid Boundary

The arrangement of the particles affects the interaction between the fluid and solid particles, and the relaxation of the solid phase can make the results more accurate. The solid particles are filled within the specific region randomly. After that, the particles are relaxed, and the particles that move out of the region will be pushed back manually using the level-set method [48,49]. Finally, the relaxed solid particles can be obtained, and the result is shown in Section 3.1.

## 3. Results and Discussion

In Section 3.1, the validation of the SPH method is verified on the two-phase problems. The sensitivity of the porous media’s structure is tested in Section 3.2. Section 3.3 presents the phase behaviors of the gas and liquid phases in heterogeneous porous media.

### 3.1. The Validation of the Scheme

**Figure 1:**
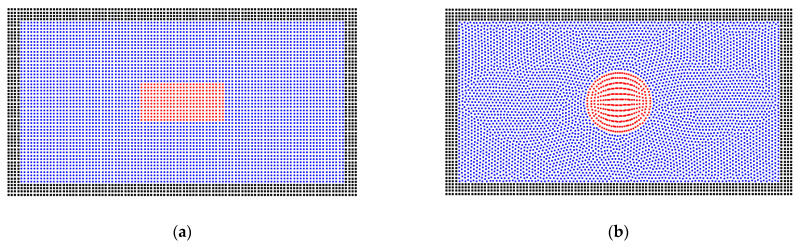
The (**a**) initial state and (**b**) equilibrium state for the gas–liquid phases in the center of the system. The red particles represent the liquid phase, the blue particles represent the gas phase, and the black particles are solid wall particles. The size of the box is 2 cm × 1 cm.

In order to verify the method for the two-phase problem of the gas and liquid, two simple cases are studied without gravity. The realistic three-dimensional porous media model is not adopted because the computational resources are huge for the three-dimensional cases. Although the realistic porous media model can present realistic results, the regular model can show the validation of the method more clearly. As shown in Figure 1, the solid particles are adopted to build a box for the simulation, and the gas and liquid particles are filled within the box. The size of the box is 2 cm × 1 cm. The initial distribution of the liquid particles is rectangular. With the effect of the surface tension between the two phases, the liquid phase tends to form the shape of a droplet, and the particles show a good arrangement on the interface, presenting a good match with previous studies [50,51]. The properties of the gas and liquid phases are shown in Table 1.

**Figure 2:**
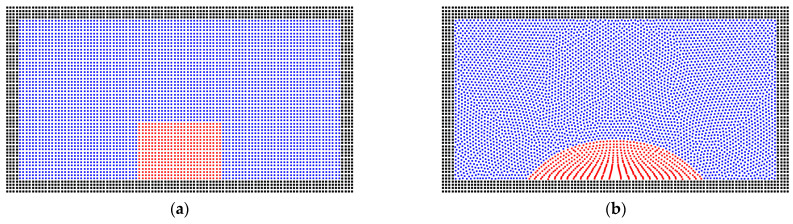

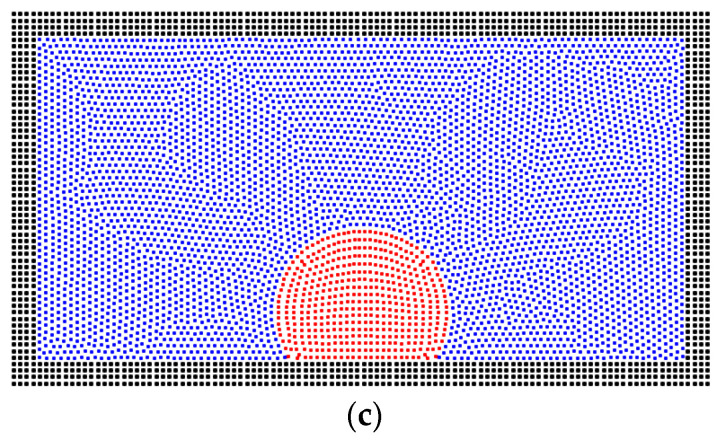
The (**a**) initial state and equilibrium states with (**b**) the contact angle of 50° and (**c**) 130° for the gas–liquid phases on the wall’s surface.

The contact angle between the liquid droplet and solid wall is also verified in the same condition, as seen in Figure 2. The liquid particles are filled at the bottom of the box with a rectangular shape. After the equilibrium simulation, the liquid phase formed a wetting droplet on the wall’s surface with the contact angle of 50°, which has a good match with the preset value. Figure 2c presents a non-wetting case with a contact angle of 130º. These two basic cases show the good validation of the SPH method on the two-phase problem.

**Figure 3:**
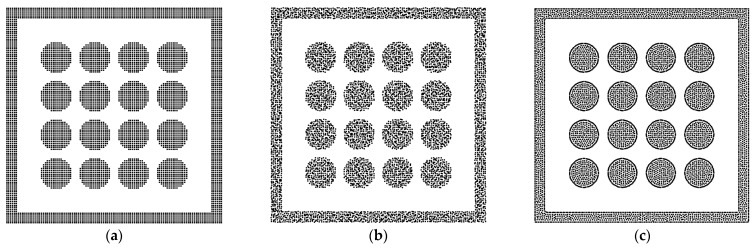
(**a**) The regular arrangement of solid particles. (**b**) The initial state before relaxing and (**c**) the relaxed state.

In addition, the solid phase is relaxed using the level-set method [49] because the arrangement of solid particles affects the fluid–solid interactions, such as the solid structure in Figure 3a. As shown in Figure 3b, the solid particles are packed randomly, and the solid particles are relaxed within the solid region [52,53]. Finally, the relaxed solid structure can be obtained in Figure 3c.

### 3.2. The Sensitivity of the Porous Media’s Structure for the Two-Phase Behavior

**Figure 4:**
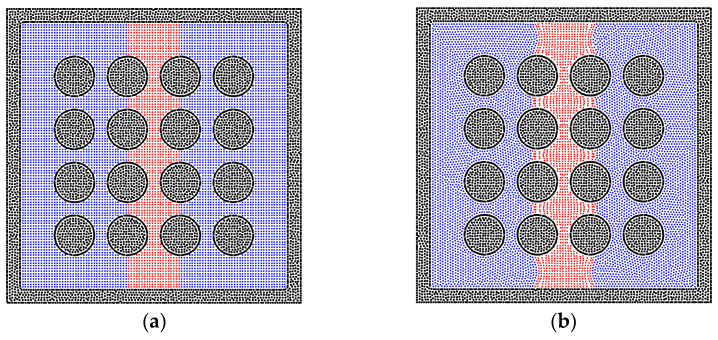
The phase distributions of the gas and liquid phases in the homogeneous porous media (contact angle = 50°). The (**a**) initial state and (**b**) equilibrium state for a liquid bridge are depicted in the system.

As shown in Figure 3, a simple porous media model is built with a number of solid spheres to represent the rock matrixes. The common states for the liquid in the rock pores are the liquid bridge and the liquid cluster. Therefore, in order to examine the effects of porous media on the imbibition problem, different initial liquid states are adopted in this study. Figure 4a presents an initial stripe state for the liquid phase, and the equilibrium state is shown in Figure 4b. In the wetting condition, the phenomenon of liquid bridge states can be observed [54]. In porous media, the liquid phase, with the effect of the surface tension, tends to move into the pore throat between the solid matrixes. Due to the homogeneity of the porous media, the liquid phase is in a balanced condition and cannot transport across the pore throat. The curvature of the matrix is also a reason why transportation is inhibited. Because the smallest pore size is in the center of the pore channel, the pore size tends to be larger if the liquid particles move out from the center position.

**Figure 5:**
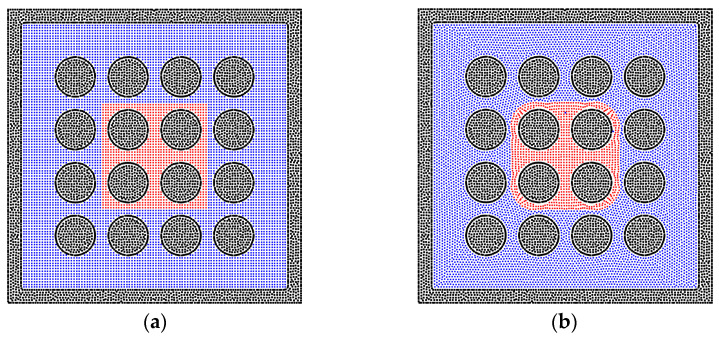
The phase distributions of the gas and liquid phases in the homogeneous porous media (contact angle = 50°). The (**a**) initial state and (**b**) equilibrium state for a liquid cluster are depicted in the system.

Apart from the liquid bridge state, the cluster state is also common in porous media. In order to judge the effect of solid matrixes accurately, the edges of the initial region are defined at the centers between different solid matrixes, as shown in Figure 5a. According to the results in Section 3.1, the liquid phase tends to be a sphere droplet in the center of the domain. However, because of the effects of the porous media and surface tension, the liquid phase still shows a smoothed square shape. Furthermore, the particles on the solid matrix’s surface tend to be taken apart by the surface tension, but the sphere shape of the solid matrix maintains the stability of the liquid film relatively.

**Figure 6:**
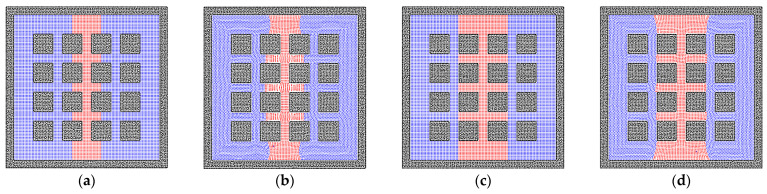
The phase distributions of the gas and liquid phases in the homogeneous porous media (contact angle = 50°), where the solid matrixes are represented by square solid particles. The (**a**) initial state and (**b**) equilibrium state for a liquid bridge are depicted in the system. The (**c**) initial state and (**d**) equilibrium state for the wider liquid bridge are also depicted in the system.

**Figure 7:**
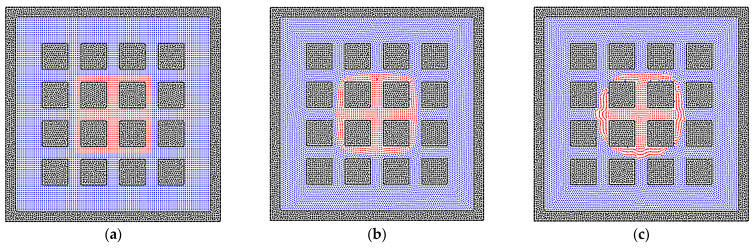
The phase distributions of the gas and liquid phases in the homogeneous porous media (contact angle = 50°), where the solid matrixes are represented by square solid particles. The (**a**) initial state, (**b**) transition state, and (**c**) equilibrium state for a liquid cluster are depicted in the system.

**Figure 8:**
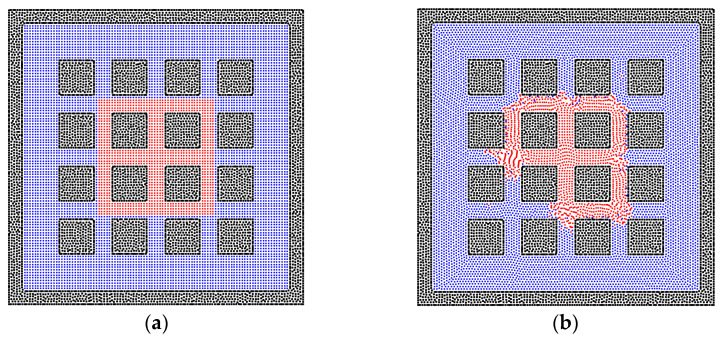
The phase distributions of the gas and liquid phases in the homogeneous porous media (contact angle = 50°), where the solid matrixes are represented by square solid particles. The (**a**) initial state and (**b**) equilibrium state for a larger liquid cluster are depicted in the system.

**Figure 9:**
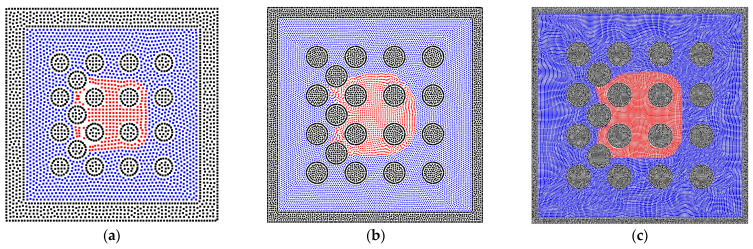
The phase distributions of the gas and liquid phases in the homogeneous porous media (contact angle = 50°) with various resolutions, (**a**) 50 × 50, (**b**) 100 × 100, and (**c**) 200 × 200.

There are various structures of rock matrixes in the reservoir. To test the effects of the solid structures clearly, the square shape of the matrix is studied in this section. The bridge and cluster liquid states are constructed initially, which are the same as that in the sphere matrix system. Figure 6b depicts the equilibrium state of the liquid bridge, which corresponds to the state in Figure 4b, but the liquid phase can go further into the square pore throat than the throat with the curvature because the throat size is constant. In Figure 6d, the wider liquid bridge is also tested, and the trapezoidal shape of the liquid bridge can be observed due to the edge effect of the square solid matrix [55]. In addition, the results of liquid cluster distributions are presented in Figure 7. The main difference is that the liquid cluster is separated at the positions of sharp corners, which is caused by the surface tension. As exhibited in Figure 7c, the separated liquid particles can move into the pore throat. Therefore, the pore throat with the curvature can block the fluid flow more easily than the square pore throat. In addition, as shown in Figure 8, the larger initial liquid cluster tends to invade the pore throats as a result of the wetting boundary condition. The particle resolution independence test is also presented in Figure 9, and the results show that the imbibition phenomenon addressed by the SPH method is relatively independent of resolutions. Figure 9a,c exhibit the imbibition trends toward the smaller pore space.

### 3.3. The Two-Phase Behavior in the Heterogeneous Porous Media

**Figure 10:**
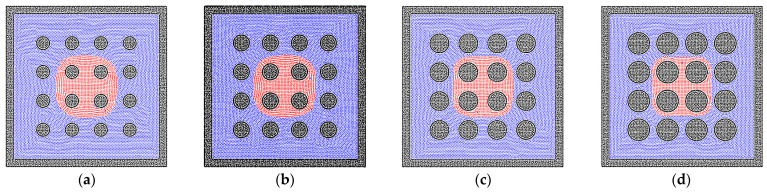
The equilibrium state for the liquid cluster in the homogeneous porous media (contact angle = 50°), and (**a**) R = 0.5 mm, (**b**) R = 0.6 mm, (**c**) R = 0.7 mm, (**d**) R = 0.8 mm, where R is the radius of each sphere wall.

**Figure 11:**
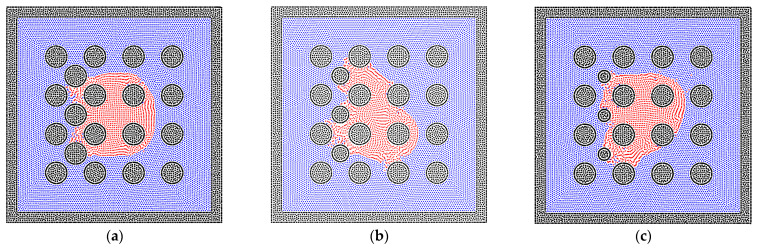
The phase distributions of the gas and liquid phases in the heterogeneous porous media (R = 0.6 mm, contact angle = 50°), where the solid matrixes with different sizes are filled in the bulk pores on the left side, and (**a**) r = 0.6 mm, (**b**) r = 0.48 mm, (**c**) r = 0.36 mm, where r is the radius of each inserted sphere wall.

**Figure 12:**
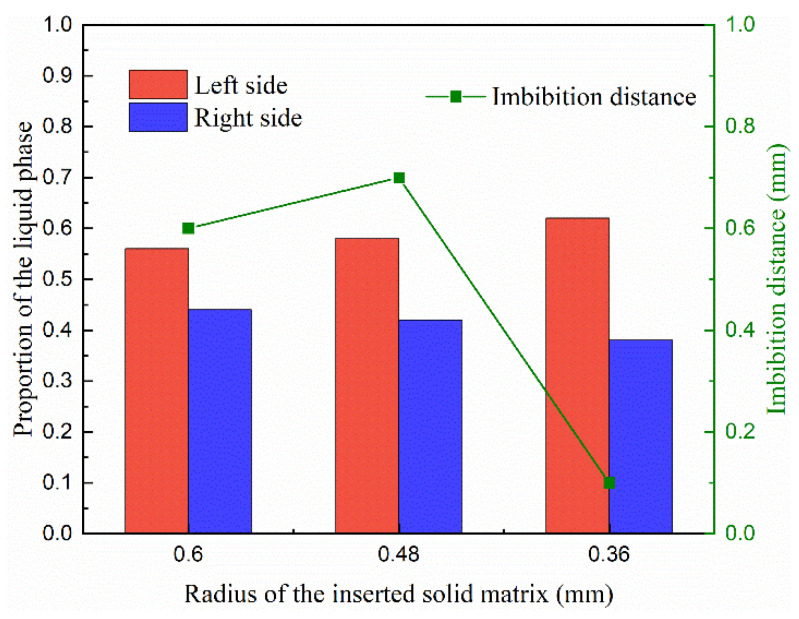
The proportion of the liquid phase distribution at the left and right sides and the imbibition distance of the liquid phase in the cases with different inserted solid matrix radii.

The pore network in porous media is usually heterogeneous, especially in the unconventional reservoir [56], and the heterogeneity of porous media is performed by the solid matrix with different sizes, inducing the different phase distributions of fluid particles. As depicted in Figure 10, the radii of solid matrixes do not change the phase distributions much in the homogeneous porous media. Therefore, the solid matrixes with different radii are inserted in the bulk of pores, as shown in Figure 11, in order to test the results in heterogeneous porous media. The imbibition effect is stronger in the results in Figure 11a,b, where the liquid phase particles already move around the inserted solid matrixes. However, in Figure 11c, the liquid phase is stopped at the position of the inserted solid matrixes because the smaller radius induces the larger pore size, which weakens the effect of interfacial tension of the liquid phase. The quantitative results are also presented in Figure 12.

**Figure 13:**
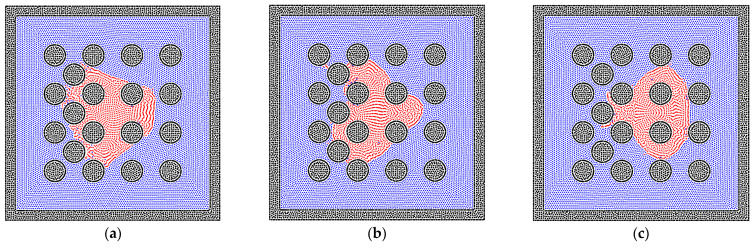
The phase distributions of the gas and liquid phases in the heterogeneous porous media (R = 0.6 mm), where the different contact angles are examined, and (**a**) contact angle = 30°, (**b**) contact angle = 90°, (**c**) contact angle = 150°.

**Figure 14:**
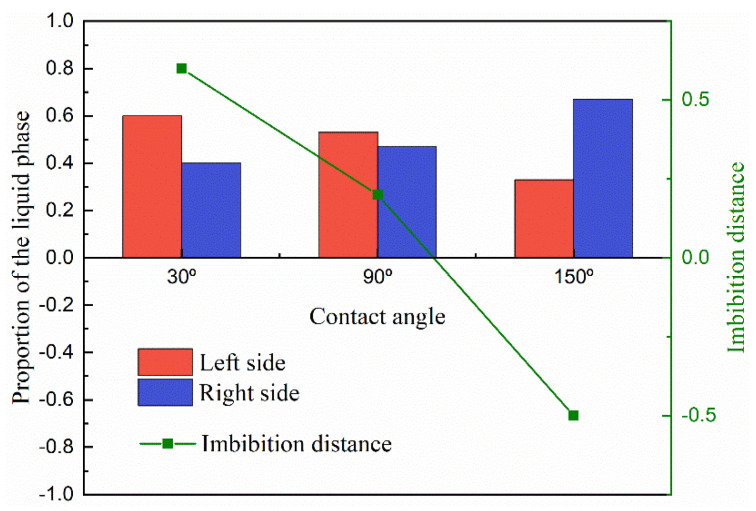
The proportion of the liquid phase distribution at the left and right sides and the imbibition distance of the liquid phase in the cases with different contact angles.

Apart from the size of the solid matrix, the contact angle is also a key point in studying the phase distribution in heterogeneous porous media. Hence, different contact angles between the liquid and solid phases are adopted and tested, which correspond to the condition of wetting, neutral, and non-wetting boundaries. As shown in Figure 13a, the liquid phase can easily perform the phenomenon of imbibition within the wetting system. In the neutral system, the imbibition of the liquid phase happens with the effect of the surface tension, but the preference of the pore size cannot be judged. Figure 13c shows the distribution of the liquid and gas phases in the non-wetting system; the liquid phase is excluded from the dense part of porous media, as shown in Figure 14. This also corresponds to previous studies because the liquid phase turns into the non-wetting phase in this condition [57,58,59].

## 4. Conclusions

In this work, the SPH method is adopted to study the gas–liquid imbibition problem in porous media. Firstly, the porous media model is built by solid particles, and the liquid and gas particles are filled in the model regularly. The SPH algorithm is verified by the basic gas–liquid models in the wetting and non-wetting systems, and the validation of the surface tension is also confirmed with a droplet model. Due to the complexity of reservoirs, the sensitivity of the porous media’s structure is examined, and the solid boundary with the curvature tends to inhibit the imbibition of the liquid phase. To mimic the heterogeneity of reservoirs, the heterogeneous porous media model is built, and the effects of the solid matrix’s size and contact angle are also tested. The smaller pore size facilitates the imbibition of the liquid phase, and the wetting solid boundary for the liquid phase contributes to the imbibition process. In contrast, the non-wetting solid boundary makes the gas the wetting phase, and the process is inversed accordingly. The imbibition behavior simulated by the SPH method is meaningful for understanding the development of oil and gas reservoirs. In this study, the applications of the SPH method in the multi-phase cases still have some problems, such as volume expansion and interface tracking, which are the points to address in our future research.

## Figures and Tables

**Table 1:** The parameters of the gas and liquid phases.

Phase	Density/(kg·m^−3^)	Viscosity/(mPa·s)
Gas	1.225	0.019
Liquid	1000	0.925

## Data Availability

Not applicable.
